# Observational Study of Topical Imiquimod Immunotherapy in the Treatment of Difficult Lentigo Maligna

**DOI:** 10.4137/cmo.s690

**Published:** 2008-12-03

**Authors:** E.E. Craythorne, C.M. Lawrence

**Affiliations:** Dept. of Dermatology, Royal Victoria Infirmary, Newcastle Upon Tyne. N1 4LP

## Introduction

Lentigo Maligna (LM) typically presents at sun-exposed sites in elderly patients as an asymmetric, slow-growing, irregularly pigmented macule with an irregular indented border. With changes in sun exposure behaviour LM is becoming more prevalent in middle-aged and younger adults.^1^

The risk of melanoma developing in LM increases with duration of the disease and therefore age,^2^ and is difficult to quantify, estimates range from 2.2%^2^ to 50%^3^ over a lifetime, once invasive disease is established, the tumour carries the same prognosis as other types of cutaneous malignant melanoma as defined by Breslow thickness and presence of ulceration.

LM presents a difficult clinical problem and generally there is little consensus on the optimum form of management. The recommended guidelines for treatment of LM are surgical excision with excision margins into clinically normal skin of 2–5 mm.^4^,^5^ Others have suggested that up to 20% of cases of LM would require margins greater than this.^6^ However, lesions are commonly large and located on cosmetically important head and neck sites. Excision may result in a poor cosmetic outcome and it is difficult to justify this approach when the risk of malignant transformation may well be low. Amelanotic lentigo maligna also remains a significant problem and inevitably results in incomplete excision since clinical identification of disease extent is impossible.^7^

Non-surgical treatment options are used in 50% of U.K. patients over the age of 70, these include radiotherapy, retinoids,^8^ 5-FU and azelaic acid. Cryotherapy, once popular, has fallen from favour because of reports of invasive melanoma occurring after cryotherapy ablation.^9^ Commonly a “watch and wait” policy is adopted.

Imiquimod (Aldara, Meda Pharmaceuticals Ltd) is a topical immune response modifier. The drug causes an increase in interferon locally and therefore may have a place in the management of superficial interferon sensitive malignancy. There are several reports of its use to treat lentigo maligna.^10^–^12^ We describe our experience with the use of imiquimod in a group of patients with LM on the head and neck where surgery was not an option.

## Patients and Methods

8 patients with histologically confirmed recurrent or difficult to treat LM of the head and neck were treated. All had declined surgery. Four LMs had recurred after previous surgery, one recurred after cryotherapy (Table 1) and three others had previously untreated large lesions with indistinct margins.

Patients were instructed to apply the cream to the pigmented area and to a minimum 1 cm margin around the pigmented area once a day, five days per week for a minimum of 6 weeks. Patients were told to expect a brisk inflammatory reaction to occur at the treated sites and to continue imiquimod despite this. At the end of the 6-week period they were reviewed.

Inflammatory response was graded as no reaction, minimal reaction, brisk reaction, very brisk reaction and was carried out by the same clinician. In some cases, where the response appeared to be minimal or there was concern that the patient had not used the cream appropriately, a further course was prescribed. At the end of therapy if pigmentation persisted this area was biopsied. Clinical photographs were taken before and after imiquimod treatment.

## Results

The clinical and demographic data for all 8 patients is given in Table 1.

8 patients (average age 67.25 years) with a total of 8 lesions of LM were treated with imiquimod. Patients were followed up until their most recent appointment, which for those responsive to treatment is an average of 34.2 months (10, 63).

## Patient Response to Imiquimod

In 3 patients (patients 1,2,8) the initial inflammatory response was minimal. Five patients (patients 3,4,5,6,7) developed a rapid and brisk response to imiquimod on first course application. The area became red, weeping and finally crusted over.

Patient 1 cleared the LM from the tip of the nose but developed pigmentation on the upper lip at a site where imiquimod had not been applied. Initially she had applied the cream very sparingly to a small area on the tip of the nose. There had been a minimal inflammatory response. She was happy with the outcome and declined a second course of treatment. Patient 2, despite apparent correct application over 2 courses, did not develop any inflammatory response. It was thought likely that she had not used sufficient cream because of her concerns about the described inflammatory reaction. The pigmented area reduced but did not resolve. Biopsy confirmed the presence of lentigo maligna. Patient 8 applied imiquimod for 6 weeks but there was no inflammatory reaction and no change in the appearance of the LM. It was subsequently discovered that he had been using a sunscreen and skin moisturiser when he had used the imiquimod. He stopped using the skin care products and reapplied the imiquimod, once a day, 5 days per week for 6 weeks; there was a very brisk response and the pigment resolved.

All of the six patients (patients 3,4,5,6,7,8) who developed an inflammatory response had clinical resolution of their LM. Patients with a clinical response were followed up for an average of 34.2 months without clinical recurrence. Patients 1 and 2 were deemed treatment failures, with the development of biopsy proven recurrence or incomplete clearance. Photography shows the brisk inflammatory reaction and clinical resolution of the LM in patients. In patient 3 (picture 3) there was residual pigmentation for 2 months and this area was biopsied. The biopsy showed pigment laden macrophages and no evidence of recurrent LM. This pigmentation resolved completely 4 months later.

## Comments

We have shown that in 6 out of 8 patients with lentigo maligna treated with imiquimod, the tumour resolved clinically with no evidence of recurrence after a mean follow up of 34.2 months. A brisk inflammatory reaction was a prerequisite of therapeutic response.

Imiquimod is a member of the imidazoquinolone drugs, a group of drugs that are unique in having both the properties of immune response modification and stimulation. It acts as an immune modulator by binding to Toll-Like Receptor 7 (TLR-7) present on macrophages, monocytes and dendrites cells. Activation of these cells leads to the release of proinflammatory mediators that drive the activation of the TH-1 pathway and upregulation of NK-cell activity. Topical application has also been shown to enhance maturation and migration of Langerhan’s cells and provide a more specific immune response.^13^ It has also been shown that imiquimod can induce apoptosis of different call lines by FasR induction, downregulation of Bcl-2, an antiapoptotic molecule, and activation of caspases 3 and 9.^14^ This may explain why it has been effective in the treatment of malignant proliferations.

Our study shows that 75% patients with LM who developed an inflammatory response to imiquimod were clinicall clear of their LM during the period of observation. Several studies^11^,^18^ have shown similar benefits, Naylor et al.^19^ demonstrated clinical and histological resolution of 93% of 28 LM 4 weeks after a 12 week treatment regimen with 80% having no evidence of relapse after a year.

Our observations support the concept that an inflammatory response is an essential component of imiquimod treatment success. Patients and physicians should be prepared for the potentially severe inflammatory response and know how to manage this. Photographs of typical reactions are useful for informing potential patients. It is also important to be aware that there will be inflammation of any co-existing actinic keratoses. In our study all patients managed to complete the course of treatment although some required a rest period because of tissue reaction. It is possible that an altered dose regimen may reduce the impact of the inflammatory response without changing the therapeutic effect.^15^

The time frame of treatment in most our patients was only for 6 weeks, 1 patient had two 6 week treatment courses. Other studies have used treatment times of up to 12 weeks. It seems that it is the inflammatory response that is important and the time taken to achieve that in most cases was a 6 weeks course.

Despite concerns that the use of imiquimod may promote tumourigeneseis and increase the rate of transformation to LMM only one case has been reported.^17^ Systemic side effects can also occur with a flu like illness, shooting pains and general malaise, and although this was not directly reported in this group these symptoms have been seen in other patients using imiquimod by the observer.^20^

Although based on only one case we have demonstrated that emollients and other skincare products may act as a barrier preventing imiquimod absorption or cause its degradation. It may be useful top warn patients not to use any other skin care product during the treatment phase.

We did not biopsy clinically cleared LM, only areas of suspicion. A pilot study^16^ showed that 5 out of 6 patients treated with LM had complete or almost complete clinical clearance despite histological persistence. If this is correct then a post-operative sample biopsy could easily miss patchy persistent disease and only complete excision of the previously affected area will be confirmatory. It is for this reason that random biopsy samples were not taken of clinically normal skin in our patients. We were unable to perform a full excision of the lesion as the recruited patients were all by definition not suitable for surgery. We do appreciate that in the absence of histological confirmations and the small numbers in the study the conclusions are softened.

LM recurrence following surgery is presumably the result of pre-existing amelanotic melanoma obscuring tumour margins and leading to incomplete excision. Thus imiquimod treatment of recurrent lesions must include a wide margin of apparently uninvolved skin around the recurrent lesion.^18^ Follow up should continue for each patient for 5 years.^21^

## Figures and Tables

**Table 1:** Patient clinical details.

	Initials	Age	Site	Status	Previous treatment	Duration of imiquimod (no. of applications per week)	Response to imiquimod	Follow up period	Disease reponse to imiquimod
Patient 1	AM	92	Nose	Recurrence	Cryotherapy	6 × 2 (7)	No response	4 months	Recurrance on biopsy
Patient 2	JJ	84	Nose	Recurrence	Surgical excision	6 × 1 (5)	minimal reaction	7 months	Recurrance on biopsy
Patient 3	MD	71	Ear Lobe	Recurrence	Surgical excision	7 × 1 (7)	Brisk response	63 months	Retained pigment in macrophagesbut no disease on biopsy
Patient 4	JS	81	Scalp	New	--------------	7 × 1 (7)	Brisk response	48 months	Clinically clear
Patient 5	EW	70	Cheek	Recurrence	Surgical excision (Mohs)	6 × 1 (5)	Very Brisk response	39 months	Clinically clear
Patient 6	IH	73	Nose	Recurrence	Surgical excision	6 × 1 (5)	Very Brisk response	24 months	Clinically clear
Patient 7	SF	87	Cheek	New	--------------	6 × 1 (5)	Brisk response	21 months	Clinically clear
Patient 8	DL First trial	50	Forehead	New	--------------	6 × 1 (5)	No response	2 months	no response
	DL Second Trial	50	Forehead			6 × 1 (5)	Brisk response	10 months	Clinically clear
